# Epigenetic regulation of the oxytocin receptor gene: implications for behavioral neuroscience

**DOI:** 10.3389/fnins.2013.00083

**Published:** 2013-05-23

**Authors:** Robert Kumsta, Elisabeth Hummel, Frances S. Chen, Markus Heinrichs

**Affiliations:** ^1^Laboratory for Biological and Personality Psychology, Department of Psychology, University of FreiburgFreiburg, Germany; ^2^Freiburg Brain Imaging Center, University Medical Center, University of FreiburgFreiburg, Germany

**Keywords:** oxytocin receptor gene, methylation, epigenetics, autistic disorder, social neuroscience

## Abstract

Genetic approaches have improved our understanding of the neurobiological basis of social behavior and cognition. For instance, common polymorphisms of genes involved in oxytocin signaling have been associated with sociobehavioral phenotypes in healthy samples as well as in subjects with mental disorders. More recently, attention has been drawn to epigenetic mechanisms, which regulate genetic function and expression without changes to the underlying DNA sequence. We provide an overview of the functional importance of oxytocin receptor gene (*OXTR*) promoter methylation and summarize studies that have investigated the role of *OXTR* methylation in behavioral phenotypes. There is first evidence that *OXTR* methylation is associated with autism, high callous-unemotional traits, and differential activation of brain regions involved in social perception. Furthermore, psychosocial stress exposure might dynamically regulate *OXTR*. Given evidence that epigenetic states of genes can be modified by experiences, especially those occurring in sensitive periods early in development, we conclude with a discussion on the effects of traumatic experience on the developing oxytocin system. Epigenetic modification of genes involved in oxytocin signaling might be involved in the mechanisms mediating the long-term influence of early adverse experiences on socio-behavioral outcomes.

## Introduction

Research across species has shown that the neuropeptide oxytocin plays a key role in the regulation of social cognition and behavior. It plays a crucial role in attachment, social exploration, and social recognition, as well as anxiety and stress-related behaviors (Meyer-Lindenberg et al., 2011). Based on oxytocin administration studies and measurements of peripheral oxytocin levels, it has been suggested that signaling of oxytocin is impaired in mental disorders associated with social deficits, including autism (Andari et al., 2010; Guastella et al., 2010), borderline personality disorder (Simeon et al., 2011), and social anxiety disorder (Guastella et al., 2009; Labuschagne et al., 2010).

Considerable progress has been made in delineating the neurobiological basis of social behavior. One avenue of research in the social neurosciences aims at identifying variations in specific genes which contribute to individual differences in social behavior and cognition, and to disease susceptibility for neuropsychiatric or developmental disorders characterized by social deficits. Several association studies have shown that genes involved in oxytocinergic signaling are important in explaining individual differences in sociobehavioral phenotypes in both healthy samples and patient groups.

The most extensively studied candidate is the gene coding for the oxytocin receptor (*OXTR*, for review, see Kumsta and Heinrichs, 2013), but other oxytocin pathway genes such as *CD38* (Jin et al., 2007; Lerer et al., 2010), and the gene coding for oxytocin itself (*OXT*; coding for the precursor protein oxytocin-neurophysin-I), have shown associations with social cognition and autism (Ebstein et al., 2009; Love et al., 2012). Regarding sociobehavioral phenotypes, single nucleotide polymorphisms (SNPs) in *OXTR* have been associated with empathy (Rodrigues et al., 2009), positive affect (Kogan et al., 2011; Montag et al., 2011) and sensitivity to social support (Chen et al., 2011). Imaging genetic studies show that *OXTR* SNPs are associated with structural and functional alterations in limbic circuitry involving the amygdala, the hypothalamus and the cingulate gyrus, suggesting that variation of *OXTR* influences social cognition and behavior by modulating neural circuits for processing of social information and negative affect (Meyer-Lindenberg and Tost, 2012).

Taken together, these studies highlight the importance of *OXTR* variation in explaining phenotypic variability of social behavior and disease susceptibility. It is worth noting, however, that the effect sizes of single SNPs are usually small. Thus, in addition to genetic studies, which are concerned with effects due to direct alterations of the DNA sequence, other factors that influence gene expression should be taken into account.

One such additional layer of genetic information that has recently become the target of considerable interest is epigenetic regulation of gene function. Epigenetics describes changes in gene activity or function which can be transmitted to the next cell generation but that occur in the absence of changes to the DNA sequence. Several mechanisms involved in the control of gene expression have been described, including DNA methylation, chromatin modification, and control of mRNA expression by non-coding RNAs, especially miRNAs (Jaenisch and Bird, 2003; Zhou et al., 2011). Most epigenetic studies in neuropsychiatry and epidemiology focus on DNA methylation, which involves direct chemical modification of the DNA, i.e., methylation of, in most cases, cytosines in cytosine-guanine (CpG) dinucleotides. In concert with other regulators, DNA methylation is recognized as an important epigenetic factor influencing gene expression (Moore et al., 2013).

Historically, DNA methylation has been recognized for its role in cellular differentiation and imprinting, mediating the distinct gene expression profiles in the multitude of cells in complex organisms. Recently, research has shown that epigenetic modifications are more pliable than previously assumed. Indeed, the epigenome seems sensitive to a wide variety of environmental influences, including diet, toxins, and maternal care (Zhang et al., 2010; Walker and Gore, 2011; Dominguez-Salas et al., 2012). Epigenetics has thus been embraced by behavioral and developmental neuroscientists as a biological mechanism for the link between environmental influences and persisting changes in physiology and behavior.

This review describes the functional importance of *OXTR* promoter methylation with regard to transcriptional control and summarizes studies that have investigated the role of *OXTR* methylation in behavioral phenotypes. There is first evidence that *OXTR* methylation is associated with autism, high callous-unemotional (CU) traits, and differential activation of brain regions involved in social perception. Furthermore, there is tentative evidence that *OXTR* methylation may be dynamically regulated by psychosocial stress exposure.

Given evidence that epigenetic states of genes can be modified by experiences, especially those occurring in sensitive periods early in development, we conclude with a discussion on the effects of traumatic experience on the developing oxytocin system. We provide an outline for future research efforts to investigate the role that epigenetics plays in mediating the long-term influence of early adverse experiences on sociobehavioral outcomes.

## Functional significance of *OXTR* DNA methylation

**Figure 1:**
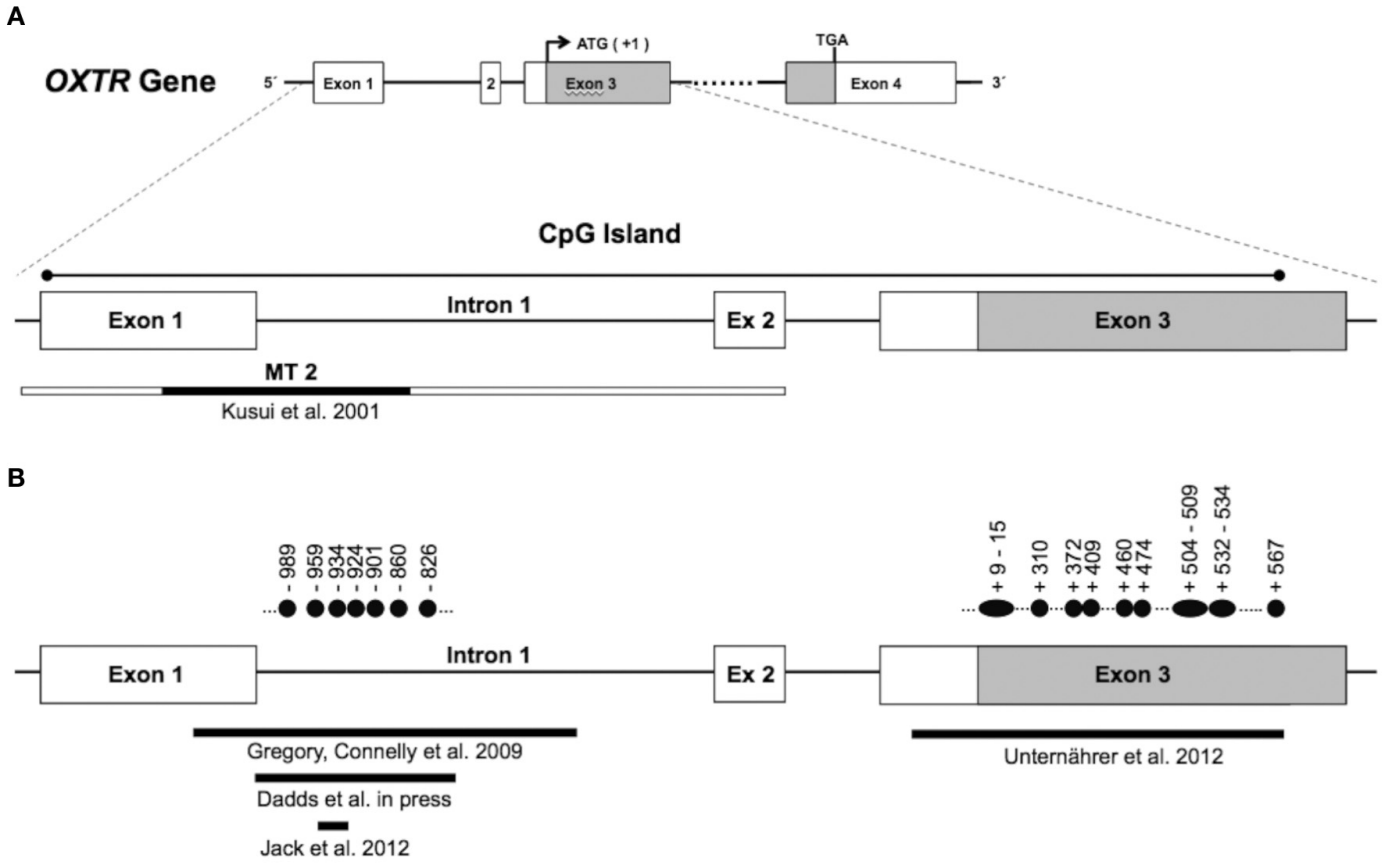
**Panel (A) (top) shows the genomic organization of the oxytocin receptor gene (*OXTR*).** The *OXTR* gene is located on chromosome 3p25–3p26.2, spans 17 kb, and contains three introns and four exons, indicated by boxes. The protein-coding region is indicated in gray (ATG denotes the transcription start site, and TGA denotes the stop codon). The enlarged section at the bottom of panel **(A)** shows the location of a CpG island, which spans exon 1 through exon 3. The genomic region investigated by Kusui et al. with regard to methylation effects on gene expression is indicated by the narrow box. The MT2 region in particular (shown in black) was shown to be functionally significant for *OXTR* gene regulation (see text). Panel **(B)** shows the regions within the CpG island that were investigated with regard to differential methylation. Filled circles indicate individual CpG sites with significant differences in methylation levels. Numbering is relative to the translation start site (+1).

In mammalian cells, the majority of DNA methylation occurs on cytosines (C) that precede a guanine (G) nucleotide, referred to as CpG sites. Certain areas of the genome contain regions of high CpG density. These regions, called “CpG islands,” are defined as a >200 bp region with GC content of more than 50% and an observed/predicted CpG ratio of more than 0.6 (Gardiner-Garden and Frommer, 1987). In the OXTR gene, there is CpG island that stretches from about 20 to 2350 bp downstream of the transcription start site (chr3:8808962–8811280: GRCh37/hg19; see Figure 1A). CpG islands often span the promoter region of genes and are associated with active gene expression (Saxonov et al., 2006). These stretches of DNA have a higher CpG density than the rest of the genome and tend to be unmethylated (Bird et al., 1985). However, when methylated, CpG islands in gene promoters contribute to transcriptional repression in most tissues (Razin, 1998).

Kusui and colleagues (2001) investigated whether methylation of the *OXTR* CpG island influenced *OXTR* transcription. Using a luciferase reporter gene assay, it was shown that the CpG island had significant promoter activity. Transcriptional activity of an unmethylated reporter construct including the CpG island (−2860 to +1342 bp relative to the transcription start site) was about 2-fold higher compared to the construct including the core promoter but lacking the majority of the CpG island (−2860 to +144 bp). Following methylation, transcriptional activity of the reporter gene construct lacking the CpG island was reduced by 19%, whereas activity of the construct including the CpG island was suppressed by 70% when methylated. This indicates that *OXTR* CpG island methylation functionally suppresses transcription, at least in the investigated hepatoblastoma cell line.

Importantly, Kusui et al. identified a region of the *OXTR* CpG island (termed MT2; see Figure 1A) that appears to be responsible for the majority of DNA methylation-induced silencing of these constructs. Deletion of the MT2 region in the full length construct led to a relative rescue of transcriptional activity of the methylated construct to 68%. This suggests that regulation of *OXTR* is sensitive to methylation within the MT2 region of the CpG island, and points toward functional significance of this region. However, the precise mechanism of how increased methylation in CpG sites of the MT2 region lead to transcriptional down-regulation of *OXTR*, and how this relates to receptor distribution in target tissue, is currently unknown and should be addressed in post-mortem investigation of brain tissue derived from healthy samples as well as patient groups.

## Autism

Several lines of evidence have implicated the oxytocin system in the etiology of autism spectrum disorder (Heinrichs et al., 2009). Whereas a number of studies have shown associations between variants of *OXTR* and autism spectrum disorder (Ebstein et al., 2012), we are aware of only one investigation assessing the role of differential methylation of *OXTR* in autism. The starting point for the study by Gregory et al. (2009) was the identification of an allelic deletion of *OXTR* in an autistic boy and his mother. The boy's affected brother, although not inheriting the deletion, showed increased methylation at two CpGs located in the MT2 region compared to the non-autistic father. This raised the possibility that methylation of *OXTR*, as a mechanism of epigenetic silencing, might be involved more generally in the etiology of autism. In a next step, analyses were extended beyond this family, and *OXTR* methylation in DNA from peripheral blood mononuclear cells (PBMCs) was investigated in 20 individuals with autism and 20 matched normotypical controls. Significantly increased methylation in autistic probands was observed for CpGs −860, −934, and −959 (numbering according to translation start site +1). Importantly, a similar pattern of methylation differences was observed in temporal cortex tissue. In an independent sample of 10 autistic and matched controls, increased methylation of CpGs −860, −901, −924, and −934, with correspondingly reduced expression of *OXTR* mRNA by 20%, was observed. Although investigated in a small sample, these findings suggest functional importance of *OXTR* promoter methylation with regard to gene regulation, and possibly the etiology of autism.

## Social perception

In addition to its role in social cognition and affiliative behavior, oxytocin seems to be involved in the processing of basic social stimuli. For instance, intranasal oxytocin administration improved emotion recognition (Domes et al., 2007b, 2010), increased covert attention to positive social cues (Domes et al., 2012), and increased time spent looking at the eye region of faces (Guastella et al., 2008).

Building on evidence of *OXTR* hypermethylation and related transcriptional differences in the temporal cortex of autistic individuals, Jack et al. (2012) investigated whether methylation of a particular CpG site (−934) might be related to activation differences in brain regions involved in biological motion perception. Functional MRI data were collected while participants (*n* = 42) passively viewed a scene in which geometrical shapes interact in ways suggestive of animacy.

Whole-brain analysis showed that individuals with higher levels of *OXTR* methylation derived from PBMCs demonstrated significantly greater activation in two clusters. The first extended from the superior temporal gyrus into supramarginal gyrus at the temporal parietal junction, and the second was in the dorsal anterior cingulate cortex (dACC). The temporal parietal junction in particular has been linked to the attributions of intentions, perception of biological motion cues, and mentalizing behaviors (Blakemore et al., 2003). These results suggest that *OXTR* methylation influences relatively low-level processes involved in social perception and may contribute more generally to interindividual differences in social cognition and behavior. Future studies should extend methylation analyses beyond the one site studied here, and go beyond investigations of neural endophenotypes in order to study the effects of methylation on social perception in naturalistic settings.

## Callous-unemotional traits

Mostly on theoretical grounds, researchers have argued for a role of oxytocin in psychopathy (Moul et al., 2012) and CU traits, considered a developmental precursor to psychopathy (Frick and White, 2008). Psychological processes that are disturbed in psychopathy, i.e., emotion recognition, empathy, and social affiliation, are all influenced by oxytocin signaling. However, apart from a few candidate gene studies associating *OXTR* SNPs with conduct problems or CU traits (Beitchman et al., 2012; Sakai et al., 2012), empirical evidence is scarce. Dadds et al. (in press) provide first evidence that *OXTR* promoter methylation is associated with CU traits in males. A subsample of 69 boys (3–16 years old) who met formal criteria for DSM-IV diagnosis of conduct problems (Oppositional Defiant Disorder or Conduct Disorder) were investigated. DNA was extracted from blood cells, and methylation levels across five CpG sites (−989, −959, −934. −924, −826) located in the MT2 area were assessed and combined into a mean methylation score. Overall, there was no significant association between methylation and CU traits. However, when taking into account age, a different pattern emerged. Whereas there was no relation between methylation and CU traits in the younger sample (3–8 years), higher methylation of *OXTR* was associated with elevated CU traits in the older sample (9–16 years).

This study did not investigate these boys longitudinally, so it is unclear whether the increased methylation levels in older boys with high CU traits reflect cumulative environmental exposure and is causally related to disturbances of social behavior, or whether *OXTR* methylation is a consequence or epiphenomenon of high CU traits caused by other factors, e.g., genetic vulnerability. This highlights the importance of longitudinal designs in epigenetic epidemiology (Wong et al., 2010).

## Psychosocial stress

In addition to its role in social behavior and social cognition, oxytocin influences the mammalian physiological stress response. It interacts both with the neuroendocrine stress response (Neumann, 2002) and with sympathetic nervous system stress reactivity (Ditzen et al., 2013). In combination with social support, intranasal administration of oxytocin has been shown to dampen neuroendocrine stress reactivity (Heinrichs et al., 2003) and to decrease amygdala activation in response to threatening stimuli (Kirsch et al., 2005; Domes et al., 2007a). Neurogenetic studies provide further evidence for the involvement of oxytocin signaling in stress reactivity (Chen et al., 2011; Tost et al., 2010).

Unternaehrer et al. (2012) investigated whether dynamic changes in *OXTR* DNA methylation would be observed after acute stress exposure. A sample of 76 participants was subjected to the Trier Social Stress Test (TSST), a standardized laboratory protocol consisting of extemporaneous public speaking and mental arithmetic tasks. Methylation levels of 35 CpG sites, located mainly in the protein coding part of exon 3 (see Figure 1B), were assessed immediately before, 1 min after, and 90 min after stress exposure. Mean methylation status increased immediately after stress, and then decreased to below baseline levels 90 min post-stress. Individual CpGs that showed significant changes across the time points are depicted by filled circles in Figure 1B.

While this investigation provides first evidence that DNA methylation status of a gene involved in stress regulation might be sensitive to acute stress exposure, these results should nevertheless be interpreted with caution. First, methylation was assessed in whole blood. As the composition of the circulating leukocyte pool can rapidly change in response to stress (Richlin et al., 2004), changes in blood cell composition might partially account for the observed differences in methylation levels. Second, the observed differences were rather small. The mean change from pre- to post-stress was 0.38, and 1.04% from post-stress to follow-up, although individual CpGs showed larger differences.

The notion of rapid changes in methylation following stress exposure is intriguing. However, in order to further investigate potential underlying mechanisms, replication studies should also include measures that might help to specify which components of the stress signaling cascades might be involved, i.e., glucocorticoid or catecholaminergic signaling, or both.

## Developmental perspectives and future directions

There is growing evidence that epigenetic states of genes can be modified by experiences, especially those occurring in sensitive periods early in development. In their seminal studies on rodents, Michael Meaney and his colleagues demonstrated a functional link between naturally occurring variations in maternal behavior and specific epigenetic modifications leading to changes in gene expression and life-long phenotypic differences in physiology and behavior, including neuroendocrine stress responsivity, fear-related behavior and attentional processes, synaptogenesis and cognitive development, female reproductive behavior and maternal care itself (for review see Zhang and Meaney, 2010). First studies are appearing that translate these findings to humans (Mcgowan et al., 2009; Labonte et al., 2012a,b).

To our knowledge, there is no published research in humans on the effects of early environmental influences on differential *OXTR* methylation. However, there is evidence that the developing central nervous oxytocin system is affected by early adversity. In a sample of adult women with a history of early abuse, decreased oxytocin concentrations in cerebrospinal fluid (CSF) were found in women reporting exposure to childhood abuse as compared to women without such experience (Heim et al., 2009).

Prolonged institutional deprivation in early childhood also seems to interfere with the developing oxytocin system. Changes in oxytocin levels after social interaction were investigated in post-institutionalized children reared in severely depriving conditions (Wismer Fries et al., 2005). Compared to children reared in a typical home environment, the adopted children showed lower peripheral oxytocin levels after physical interactions with their adoptive mothers.

It has been hypothesized that the observed long-term effects of adverse childhood experiences on deficits in social behavior and cognition (Repetti et al., 2002; Kumsta et al., 2010) might be mediated through oxytocin functioning. The observation that the effects of adverse childhood experiences last well beyond childhood and increase risk for the development of a wide variety of diseases in adulthood (Gilbert et al., 2009), points toward enduring biological effects underlying these associations and also raises the question of how these effects retain their stability. Epigenetic mechanisms potentially constitute such a mechanism, serving as a molecular link between “nurture” and “nature.” Future studies are warranted to investigate the role of epigenetic regulation of genes involved in oxytocin signaling in mediating the long-term influence of early adverse experiences on socio-behavioral outcomes.

Given the excitement surrounding epigenetics research, words of caution have been raised. For instance, all studies included here measured DNA methylation from whole blood. Since blood is a heterogeneous tissue, it is unclear to what extent DNA methylation difference between groups could be confounded by differences in the cellular composition of the samples. Another important question concerns the extent to which peripheral tissues can be used to address questions about variation in inaccessible tissues of interest, such as the brain. It is currently unknown whether differences in *OXTR* methylation obtained from peripheral tissues reflect variation in central nervous nuclei expressing OXTR. A detailed account of the necessary precautions and considerations surrounding the application of epigenetics to behavioral sciences is outside the scope of this mini-review, and the reader is referred to the paper by Heijmans and Mill (2012).

## Conclusion

The study of epigenetics has raised much excitement in the field of behavioral neuroscience, as it provides a compelling mechanism underlying the interplay between psychosocial experience and molecular processes influencing gene expression.

Differential methylation of a CpG island in the *OXTR* promoter seems to be functionally important for *OXTR* expression, and differences in the degree of methylation have been observed in childhood disorders characterized by impairments in social cognition. Furthermore, differential methylation of *OXTR* might be important in explaining individual differences in social behavior and cognition more broadly, and might provide a mechanism for biological embedding of early experience. Potentially, improved epigenetic understanding of “social disorders” might aid translational efforts to develop individualized clinical treatment approaches.

### Conflict of interest statement

The authors declare that the research was conducted in the absence of any commercial or financial relationships that could be construed as a potential conflict of interest.

## References

[B1] Andari E. Duhamel J. R. Zalla T. Herbrecht E. Leboyer M. Sirigu A. (2010). Promoting social behavior with oxytocin in high-functioning autism spectrum disorders. Proc. Natl. Acad. Sci. U.S.A. 107, 4389–4394 10.1073/pnas.0910249107 20160081 PMC2840168

[B2] Beitchman J. H. Zai C. C. Muir K. Berall L. Nowrouzi B. Choi E. (2012). Childhood aggression, callous-unemotional traits and oxytocin genes. Eur. Child Adolesc. Psychiatry 21, 125–132 10.1007/s00787-012-0240-6 22294460

[B3] Bird A. Taggart M. Frommer M. Miller O. J. Macleod D. (1985). A fraction of the mouse genome that is derived from islands of nonmethylated, CpG-rich DNA. Cell 40, 91–99 10.1016/0092-8674(85)90312-5 2981636

[B4] Blakemore S. J. Boyer P. Pachot-Clouard M. Meltzoff A. Segebarth C. Decety J. (2003). The detection of contingency and animacy from simple animations in the human brain. Cereb. Cortex 13, 837–844 10.1093/cercor/13.8.837 12853370

[B5] Chen F. S. Kumsta R. Von Dawans B. Monakhov M. Ebstein R. P. Heinrichs M. (2011). Common oxytocin receptor gene (*OXTR*) polymorphism and social support interact to reduce stress in humans. Proc. Natl. Acad. Sci. U.S.A. 108, 19937–19942 10.1073/pnas.1113079108 22123970 PMC3250137

[B6] Dadds M. R. Moul C. Cauchi A. Dobson-Stone C. Hawes D. Brennan J. (in press). Oxytocin receptor genetics, epigenetics, and the development of psychopathy. Dev. Psychopathol. 10.1017/S0954579413000485 24059750

[B7] Ditzen B. Nater U. M. Schaer M. La Marca R. Bodenmann G. Ehlert U. (2013). Sex-specific effects of intranasal oxytocin on autonomic nervous system and emotional responses to couple conflict. Soc. Cogn. Affect. Neurosci. [Epub ahead of print]. 10.1093/scan/nss083 22842905 PMC3831552

[B8] Domes G. Heinrichs M. Glascher J. Buchel C. Braus D. Herpertz S. (2007a). Oxytocin attenuates amygdala responses to emotional faces regardless of valence. Biol. Psychiatry 62, 1187–1190 10.1016/j.biopsych.2007.03.025 17617382

[B9] Domes G. Heinrichs M. Michel A. Berger C. Herpertz S. C. (2007b). Oxytocin improves “mind-reading” in humans. Biol. Psychiatry 61, 731–733 10.1016/j.biopsych.2006.07.015 17137561

[B10] Domes G. Lischke A. Berger C. Grossmann A. Hauenstein K. Heinrichs M. (2010). Effects of intranasal oxytocin on emotional face processing in women. Psychoneuroendocrinology 35, 83–93 10.1016/j.psyneuen.2009.06.016 19632787

[B11] Domes G. Sibold M. Schulze L. Lischke A. Herpertz S. C. Heinrichs M. (2012). Intranasal oxytocin increases covert attention to positive social cues. Psychol. Med. [Epub ahead of print]. 10.1017/S0033291712002565 23146328

[B12] Dominguez-Salas P. Cox S. E. Prentice A. M. Hennig B. J. Moore S. E. (2012). Maternal nutritional status, C(1) metabolism and offspring DNA methylation: a review of current evidence in human subjects. Proc. Nutr. Soc. 71, 154–165 10.1017/S0029665111003338 22124338 PMC3491641

[B13] Ebstein R. P. Israel S. Lerer E. Uzefovsky F. Shalev I. Gritsenko I. (2009). Arginine vasopressin and oxytocin modulate human social behavior. Ann. N.Y. Acad. Sci. 1167, 87–102 10.1111/j.1749-6632.2009.04541.x 19580556

[B14] Ebstein R. P. Knafo A. Mankuta D. Chew S. H. Lai P. S. (2012). The contributions of oxytocin and vasopressin pathway genes to human behavior. Horm. Behav. 61, 359–379 10.1016/j.yhbeh.2011.12.014 22245314

[B15] Frick P. J. White S. F. (2008). Research review: the importance of callous-unemotional traits for developmental models of aggressive and antisocial behavior. J. Child Psychol. Psychiatry. 49, 359–375 10.1111/j.1469-7610.2007.01862.x 18221345

[B16] Gardiner-Garden M. Frommer M. (1987). CpG islands in vertebrate genomes. J. Mol. Biol. 196, 261–282 10.1016/0022-2836(87)90689-9 3656447

[B17] Gilbert R. Widom C. S. Browne K. Fergusson D. Webb E. Janson S. (2009). Burden and consequences of child maltreatment in high-income countries. Lancet 373, 68–81 10.1016/S0140-6736(08)61706-7 19056114

[B18] Gregory S. G. Connelly J. J. Towers A. J. Johnson J. Biscocho D. Markunas C. A. (2009). Genomic and epigenetic evidence for oxytocin receptor deficiency in autism. BMC Medicine 7:62 10.1186/1741-7015-7-62 19845972 PMC2774338

[B19] Guastella A. J. Einfeld S. L. Gray K. M. Rinehart N. J. Tonge B. J. Lambert T. J. (2010). Intranasal oxytocin improves emotion recognition for youth with autism spectrum disorders. Biol. Psychiatry 67, 692–694 10.1016/j.biopsych.2009.09.020 19897177

[B20] Guastella A. J. Howard A. L. Dadds M. R. Mitchell P. Carson D. S. (2009). A randomized controlled trial of intranasal oxytocin as an adjunct to exposure therapy for social anxiety disorder. Psychoneuroendocrinology 34, 917–923 10.1016/j.psyneuen.2009.01.005 19246160

[B21] Guastella A. J. Mitchell P. B. Dadds M. R. (2008). Oxytocin increases gaze to the eye region of human faces. Biol. Psychiatry 63, 3–5 10.1016/j.biopsych.2007.06.026 17888410

[B22] Heijmans B. T. Mill J. (2012). Commentary: the seven plagues of epigenetic epidemiology. Int. J. Epidemiol. 41, 74–78 10.1093/ije/dyr225 22269254 PMC3304528

[B23] Heim C. Young L. J. Newport D. J. Mletzko T. Miller A. H. Nemeroff C. B. (2009). Lower CSF oxytocin concentrations in women with a history of childhood abuse. Mol. Psychiatry 10, 954–958 10.1038/mp.2008.112 18957940

[B24] Heinrichs M. Baumgartner T. Kirschbaum C. Ehlert U. (2003). Social support and oxytocin interact to suppress cortisol and subjective responses to psychosocial stress. Biol. Psychiatry 54, 1389–1398 10.1016/S0006-3223(03)00465-7 14675803

[B25] Heinrichs M. Von Dawans B. Domes G. (2009). Oxytocin, vasopressin, and human social behavior. Front. Neuroendocrinol. 30, 548–557 10.1016/j.yfrne.2009.05.005 19505497

[B26] Jack A. Connelly J. J. Morris J. P. (2012). DNA methylation of the oxytocin receptor gene predicts neural response to ambiguous social stimuli. Front. Hum. Neurosci. 6:280 10.3389/fnhum.2012.00280 23087634 PMC3467966

[B27] Jaenisch R. Bird A. (2003). Epigenetic regulation of gene expression: how the genome integrates intrinsic and environmental signals. Nat. Genet. 33 Suppl., 245–254 10.1038/ng1089 12610534

[B28] Jin D. Liu H. X. Hirai H. Torashima T. Nagai T. Lopatina O. (2007). CD38 is critical for social behaviour by regulating oxytocin secretion. Nature 446, 41–45 10.1038/nature05526 17287729

[B29] Kirsch P. Esslinger C. Chen Q. Mier D. Lis S. Siddhanti S. (2005). Oxytocin modulates neural circuitry for social cognition and fear in humans. J. Neurosci. 25, 11489–11493 10.1523/JNEUROSCI.3984-05.2005 16339042 PMC6725903

[B30] Kogan A. Saslow L. R. Impett E. A. Oveis C. Keltner D. Rodrigues Saturn S. (2011). Thin-slicing study of the oxytocin receptor (*OXTR*) gene and the evaluation and expression of the prosocial disposition. Proc. Natl. Acad. Sci. U.S.A. 108, 19189–19192 10.1073/pnas.1112658108 22084107 PMC3228468

[B31] Kumsta R. Heinrichs M. (2013). Oxytocin, stress and social behavior: neurogenetics of the human oxytocin system. Curr. Opin. Neurobiol. 23, 11–16 10.1016/j.conb.2012.09.004 23040540

[B32] Kumsta R. Kreppner J. Rutter M. Beckett C. Castle J. Stevens S. (2010). III. Deprivation-specific psychological patterns. Monogr. Soc. Res. Child Dev. 75, 48–78 10.1111/j.1540-5834.2010.00550.x 20500633

[B33] Kusui C. Kimura T. Ogita K. Nakamura H. Matsumura Y. Koyama M. (2001). DNA methylation of the human oxytocin receptor gene promoter regulates tissue-specific gene suppression. Biochem. Biophys. Res. Commun. 289, 681–686 10.1006/bbrc.2001.6024 11726201

[B34] Labonte B. Suderman M. Maussion G. Navaro L. Yerko V. Mahar I. (2012a). Genome-wide epigenetic regulation by early-life trauma. Arch. Gen. Psychiatry 69, 722–731 10.1001/archgenpsychiatry.2011.2287 22752237 PMC4991944

[B35] Labonte B. Yerko V. Gross J. Mechawar N. Meaney M. J. Szyf M. (2012b). Differential glucocorticoid receptor exon 1(B), 1(C), and 1(H) expression and methylation in suicide completers with a history of childhood abuse. Biol. Psychiatry 72, 41–48 10.1016/j.biopsych.2012.01.034 22444201

[B36] Labuschagne I. Phan K. L. Wood A. Angstadt M. Chua P. Heinrichs M. (2010). Oxytocin attenuates amygdala reactivity to fear in generalized social anxiety disorder. Neuropsychopharmacology 35, 2403–2413 10.1038/npp.2010.123 20720535 PMC3055328

[B37] Lerer E. Levi S. Israel S. Yaari M. Nemanov L. Mankuta D. (2010). Low CD38 expression in lymphoblastoid cells and haplotypes are both associated with autism in a family-based study. Autism Res. 3, 293–302 10.1002/aur.156 21182206

[B38] Love T. M. Enoch M. A. Hodgkinson C. A. Pecina M. Mickey B. Koeppe R. A. (2012). Oxytocin gene polymorphisms influence human dopaminergic function in a sex-dependent manner. Biol. Psychiatry 72, 198–206 10.1016/j.biopsych.2012.01.033 22418012 PMC3392442

[B39] Mcgowan P. O. Sasaki A. D'Alessio A. C. Dymov S. Labonte B. Szyf M. (2009). Epigenetic regulation of the glucocorticoid receptor in human brain associates with childhood abuse. Nat. Neurosci. 12, 342–348 10.1038/nn.2270 19234457 PMC2944040

[B40] Meyer-Lindenberg A. Domes G. Kirsch P. Heinrichs M. (2011). Oxytocin and vasopressin in the human brain: social neuropeptides for translational medicine. Nat. Rev. Neurosci. 12, 524–538 10.1038/nrn3044 21852800

[B41] Meyer-Lindenberg A. Tost H. (2012). Neural mechanisms of social risk for psychiatric disorders. Nat. Neurosci. 15, 663–668 10.1038/nn.3083 22504349

[B42] Montag C. Fiebach C. J. Kirsch P. Reuter M. (2011). Interaction of 5-HTTLPR and a variation on the oxytocin receptor gene influences negative emotionality. Biol. Psychiatry 69, 601–603 10.1016/j.biopsych.2010.10.026 21183159

[B43] Moore L. D. Le T. Fan G. (2013). DNA methylation and its basic function. Neuropsychopharmacology 38, 23–38 10.1038/npp.2012.112 22781841 PMC3521964

[B44] Moul C. Killcross S. Dadds M. R. (2012). A model of differential amygdala activation in psychopathy. Psychol. Rev. 119, 789–806 10.1037/a0029342 22800411

[B45] Neumann I. D. (2002). Involvement of the brain oxytocin system in stress coping: interactions with the hypothalamo-pituitary-adrenal axis. Prog. Brain Res. 139, 147–162 12436933 10.1016/s0079-6123(02)39014-9

[B46] Razin A. (1998). CpG methylation, chromatin structure and gene silencing-a three-way connection. EMBO J. 17, 4905–4908 10.1093/emboj/17.17.4905 9724627 PMC1170819

[B47] Repetti R. L. Taylor S. E. Seeman T. E. (2002). Risky families: family social environments and the mental and physical health of offspring. Psychol. Bull. 128, 330–366 10.1037/0033-2909.128.2.330 11931522

[B48] Richlin V. A. Arevalo J. M. Zack J. A. Cole S. W. (2004). Stress-induced enhancement of NF-kappaB DNA-binding in the peripheral blood leukocyte pool: effects of lymphocyte redistribution. Brain Behav. Immun. 18, 231–237 10.1016/j.bbi.2003.08.001 15050650

[B49] Rodrigues S. M. Saslow L. R. Garcia N. John O. P. Keltner D. (2009). Oxytocin receptor genetic variation relates to empathy and stress reactivity in humans. Proc. Natl. Acad. Sci. U.S.A. 106, 21437–21441 10.1073/pnas.0909579106 19934046 PMC2795557

[B50] Sakai J. T. Crowley T. J. Stallings M. C. McQueen M. Hewitt J. K. Hopfer C. (2012). Test of association between 10 single nucleotide polymorphisms in the oxytocin receptor gene and conduct disorder. Psychiatr. Genet. 22, 99–102 10.1097/YPG.0b013e32834c0cb2 21934640 PMC3337143

[B51] Saxonov S. Berg P. Brutlag D. L. (2006). A genome-wide analysis of CpG dinucleotides in the human genome distinguishes two distinct classes of promoters. Proc. Natl. Acad. Sci. U.S.A. 103, 1412–1417 10.1073/pnas.0510310103 16432200 PMC1345710

[B52] Simeon D. Bartz J. Hamilton H. Crystal S. Braun A. Ketay S. (2011). Oxytocin administration attenuates stress reactivity in borderline personality disorder: a pilot study. Psychoneuroendocrinology 36, 1418–1421 10.1016/j.psyneuen.2011.03.013 21546164

[B53] Tost H. Kolachana B. Hakimi S. Lemaitre H. Verchinski B. A. Mattay V. S. (2010). A common allele in the oxytocin receptor gene (*OXTR*) impacts prosocial temperament and human hypothalamic-limbic structure and function. Proc. Natl. Acad. Sci. U.S.A. 107, 13936–13941 10.1073/pnas.1003296107 20647384 PMC2922278

[B54] Unternaehrer E. Luers P. Mill J. Dempster E. Meyer A. H. Staehli S. (2012). Dynamic changes in DNA methylation of stress-associated genes (*OXTR, BDNF*) after acute psychosocial stress. Transl. Psychiatry 2, e150 10.1038/tp.2012.77 22892716 PMC3432191

[B55] Walker D. M. Gore A. C. (2011). Transgenerational neuroendocrine disruption of reproduction. Nat. Rev. Endocrinol. 7, 197–207 10.1038/nrendo.2010.215 21263448 PMC3976559

[B56] Wismer Fries A. B. Ziegler T. E. Kurian J. R. Jacoris S. Pollak S. D. (2005). Early experience in humans is associated with changes in neuropeptides critical for regulating social behavior. Proc. Natl. Acad. Sci. U.S.A. 102, 17237–17240 10.1073/pnas.0504767102 16303870 PMC1287978

[B57] Wong C. C. Caspi A. Williams B. Craig I. W. Houts R. Ambler A. (2010). A longitudinal study of epigenetic variation in twins. Epigenetics 5, 516–526 10.4161/epi.5.6.12226 20505345 PMC3322496

[B58] Zhang T. Y. Hellstrom I. C. Bagot R. C. Wen X. Diorio J. Meaney M. J. (2010). Maternal care and DNA methylation of a glutamic acid decarboxylase 1 promoter in rat hippocampus. J. Neurosci. 30, 13130–13137 10.1523/JNEUROSCI.1039-10.2010 20881131 PMC6633524

[B59] Zhang T. Y. Meaney M. J. (2010). Epigenetics and the environmental regulation of the genome and its function. Annu. Rev. Psychol. 61, 439–466, C431–C433. 10.1146/annurev.psych.60.110707.163625 19958180

[B60] Zhou V. W. Goren A. Bernstein B. E. (2011). Charting histone modifications and the functional organization of mammalian genomes. Nat. Rev. Genet. 12, 7–18 10.1038/nrg2905 21116306

